# YKL-40—A Protein in the Field of Translational Medicine: A Role as a Biomarker in Cancer Patients?

**DOI:** 10.3390/cancers2031453

**Published:** 2010-07-12

**Authors:** Nicolai A. Schultz, Julia S. Johansen

**Affiliations:** 1 Departments of Surgical Gastroenterology, Herlev Hospital, University of Copenhagen, Herlev Ringvej 75, DK-2730 Herlev, Denmark; E-Mail: nicolai-schultz@dadlnet.dk; 2 Departments of Oncology, Herlev Hospital, University of Copenhagen, Herlev Ringvej 75, DK-2730 Herlev, Denmark; 3 Departments of Medicine, Herlev Hospital, University of Copenhagen, Herlev Ringvej 75, DK-2730 Herlev, Denmark

**Keywords:** biomarker, cancer, CHI3L1, inflammation, YKL-40, tissue remodelling

## Abstract

YKL-40 is a 40 kDa glycoprotein produced by cancer cells, inflammatory cells and stem cells. It probably has a role in cell proliferation and differentiation, inflammation, protection against apoptosis, stimulation of angiogenesis, and regulation of extracellular tissue remodelling. Plasma levels of YKL-40 are often elevated in patients with localized or advanced cancer compared to age-matched healthy subjects. Several studies have demonstrated that high plasma YKL-40 is an independent prognostic biomarker of short survival in patients with different types of cancer. However, there is not yet sufficient data to support determination of plasma YKL-40 outside research projects as a biomarker for screening of gastrointestinal cancer and determination of treatment response and poor prognosis before or during treatment and follow-up. Plasma YKL-40 is also elevated in patients with other diseases than cancer, e.g., severe infections, cardiovascular disease, diabetes, chronic obstructive lung disease, asthma, liver fibrosis and rheumatoid arthritis. Co-morbidity should therefore always be considered in patients with cancer, since other sources than cancer cells can increase plasma YKL-40 levels. Future focused translational research projects combining basic and clinical research are needed in a joint effort to answer questions of the complex function and regulation of YKL-40 and the question if plasma YKL-40 is a clinical useful biomarker in patients with cancer.

## 1. Introduction

A critical step in translational medicine for personalizing treatment is to identify new diagnostic, prognostic and predictive cancer biomarkers. There has therefore been an increasing focus on identification of novel cancer biomarkers. Tumor molecular profiling is now included in many clinical trials [1]. New biomarkers have the potential to identify cancer patients at an early stage, select patients who are most likely to benefit from therapy and to guide the development of more effective agents. New biomarkers will help to personalize standard medicine for patients with cancer.

Tumor molecular heterogeneity is a major reason that patients with cancer with a similar tumor histology and clinical stage can have a very different clinical outcome and response to treatment with chemotherapy and biologics (e.g., antibodies against the epidermal growth factor receptors and insulin growth factor receptor). A new generation of molecular technologies, including genomic, proteomic and metabolomic mapping, holds the promise of translating into practice the use of biomarker panels for increased diagnostic and therapeutic sensitivity and specificity. 

A protein biomarker is, in general defined, as a measurable protein that is an indicator of normal biologic processes, pathogenic processes, and/or response to therapeutic or other interventions. The definition does not include specific technologies except that the biomarker should be possible to measure objectively [2] (http://www.fda.gov/cder/guidance/6400fnl.htm/, September 2004). Protein cancer biomarkers should have an association with cancer and an application in clinical detection (screening and early diagnosis) and management (prognosis, treatment response and monitoring). Prognostic biomarkers can forecast survival independent of the treatment administered. Predictive biomarkers should identify cancer patients who are likely to have increased sensitivity or resistance to a given therapy reflected in either tumor shrinkage or growth and prolonged survival or decreased survival. Biomarkers can be both prognostic and predictive. It is most likely that biomarkers should be combined in panels of biomarkers for optimal clinical use, since most biomarkers will probably individually lack optimal sensitivity and specificity. This review focuses on plasma YKL-40 as a potential new cancer biomarker.

## 2. YKL-40 Gene and Protein

In 1997, the human gene encoding the protein YKL-40 was isolated [3]. It is located on chromosome 1q32.1, has a size of 7948 base pairs and consists of 10 exons [3,4]. Two splice forms of the YKL-40 gene are reported; isoform 1 contains exon 1-10 and in isoform 2 exon 8 is spliced out [5]. 

YKL-40 [6] is also named human cartilage glycoprotein-39 (HC-gp39) [7], 38-kDa heparin-binding glycoprotein (gp38k) [8], chitinase-3-like-1 (CHI3L1) [3], and chondrex [9]. It is a glycoprotein and a member of "Family 18 chitolectins" [10,11,12] and “Mammalian chitinase like proteins”. YKL-40 is described in human [7], chimpanzee, pig [8], cow, goat [13], sheep, guinea pig [14], rat and mouse [15] and is phylogenetically highly conserved. The fruit fly *Drosophila* *melanogaster* [16,17], the mosquito *Anopheles gambiae*, the zebra fish *Danio rerio*, the pacific oyster *Crassostrea gigas* [18] and the nematode *Caenorhabditis elegans* have putative YKL-40-like proteins (National Center for Biotechnology Information (NCBI)). The YKL-40 protein contains a single polypeptide chain of 383 amino acids and has a molecular mass of 40 kDa [7] and an isoelectric point of 7.6 [19]. The crystallographic structure for human YKL-40 [20,21] displays the typical fold of family 18 glycosyl hydrolases [22]. 

## 3. YKL-40 in Healthy Subjects

### 3.1. Tissue

**Figure 1:**
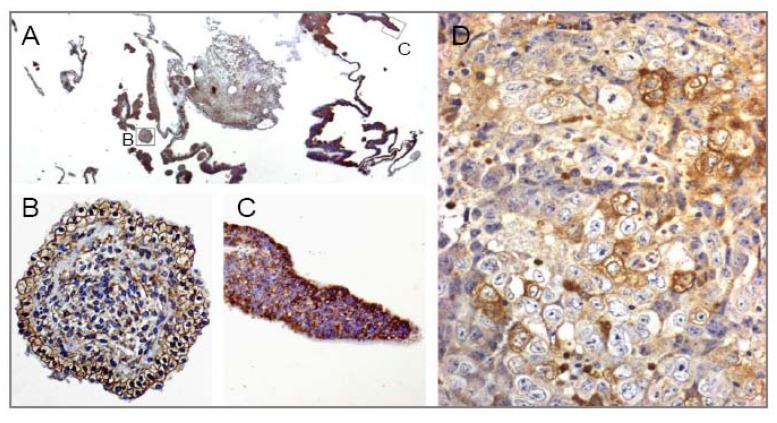
YKL-40 protein expression determined by immunohistochemical analysis in cultures of human embryonic stem cells (A) and in a biopsy of human embryonal carcinoma (D). Membrane staining of the region enclosed by square labelled B in A (B) or strong cytoplasmatic staining are seen in embryonic stem cells *in vitro* (C) and in embryonal carcinoma cells *in vivo* (D).

We have found that YKL-40 is produced by human embryonic stem cells and their progenitors, Figure 1**A-C** [23]. YKL-40 mRNA and protein expressions are also strongly expressed in all germ layers of human embryos and in human fetal tissues of ecto-, meso- and endoderm layers [5]. At the cellular level YKL-40 protein expression is high in embryonic and fetal tissues characterized by rapid proliferation and marked differentiation, and in tissues undergoing morphogenetic changes [5]. In adult normal tissue high YKL-40 expression is observed in cells with high cellular activity [24]. 

### 3.2. Plasma Concentrations of YKL-40 in Healthy Subjects

To be clinically useful determination of plasma YKL-40 must use specific, sensitive, cheap and fast methods allowing reliable measurements, optimal capability for high throughput tests, prompt turnaround time, and reasonable cost. The most used commercial enzyme-linked immunoassay (ELISA) to determine plasma YKL-40 is from Quidel (Santa Clara, CA, USA) [9]. It uses streptavidin-coated microplate wells, a biotinylated-Fab monoclonal mouse antibody against human YKL-40 (capture antibody) and an alkaline phosphatase-labeled polyclonal rabbit antibody against human YKL-40 (detection antibody). The sensitivity is 20 µg/L, detection limit 8 µg/L, and recovery 102%. The intra-assay coefficients of variations are 5% (at 40 µg/L), 4% (at 104 µg/L), and 4% (at 155 µg/L). The inter-assay coefficient of variation is <6% (personal observations). An ELISA from R&D Systems (Abingdon, Oxon, United Kingdom) is also become commercial available, but there are no publications regarding this ELISA. 

Plasma levels of YKL-40 in both sexes are highly (p < 0.001) correlated with age [9,25,26,27]. Thus, the normal, age-corrected, upper level of plasma YKL-40 is an important factor to consider in clinical studies of patients with cancer using plasma YKL-40 as a potential biomarker. There is no difference in plasma YKL-40 between the two sexes and the median plasma YKL-40 level in healthy subjects is ~40 µg/L [9,25,26]. In these three studies used to determine the normal plasma YKL-40 levels, there were no follow-up of the subjects after the blood samples were collected for plasma YKL-40 determination. This is an important problem, since it has been reported that plasma YKL-40 can be elevated years before a subject has clinical signs of cancer [27]. We have therefore determined plasma YKL-40 in a large group of 3610 healthy adults, aged 20–95 years, from the Danish general population, the Copenhagen City Heart Study. These subjects had no known disease at time of blood sampling in 1991–1994 and remained healthy during the 16 years follow-up period (Bojesen, S.E., manuscript in preparation). We found that plasma YKL-40 increased significantly with age in both sexes, and was not associated with body mass index (BMI) and plasma C-reactive protein (CRP) levels. Furthermore, plasma YKL-40 was stable in subjects that remain healthy during a 10 years period. Based on the results from this large study of truly healthy subjects we suggest that an elevated plasma YKL-40 is defined as an age-corrected plasma YKL-40 concentration higher than the 95^th^ or the 97^th^ percentile of plasma YKL-40 in healthy age-matched subjects. 

Recently, a new technique to determine plasma YKL-40 has been developed using the proximity ligation assay (PLA) [28,29]. This method employs pairs of antibodies coupled to DNA oligonucleotides such that when the antibody pairs bind to the target protein, the local concentration of DNA oligonucleotides increases to allow for enzymatic ligation of the two strands. The resulting amplicons are unique for each specific protein detected and can be measured in a highly quantitative manner by real-time quantitative real time polymerase chain reaction (qPCR). The PLA technique can be multiplexed for simultaneous detection of multiple proteins, is highly sensitive with limited background, and requires only 10 µL of plasma. This technology has been piloted in small scale biomarker studies where YKL-40 is one of 83 biomarkers [28,29]. However, more validation studies are needed and this multiplex PLA system is not yet commercially available.

A standard operating procedure must be used when handling blood samples for determination of plasma YKL-40. The time interval between drawing of blood and centrifugation of blood stored at room temperature must be less than eight hours for EDTA plasma samples and less than three hours for serum. Otherwise, significant and non-disease related elevations in plasma YKL-40 are found. If the blood is stored at 4 °C before centrifugation YKL-40 concentrations are stable in EDTA plasma for 72 hours and in serum for 24 hours [30]. Degranulation of neutrophils will release YKL-40 from the specific granules, and neutrophils are the main source of this time dependent increase in YKL-40 concentrations in serum and EDTA plasma. After the blood cells are removed, YKL-40 is stable in plasma stored up to five days at room temperature [30], up to nine days at 4 °C [9], and at −80 °C for at least 14 years (personal observation). Repetitive freezing and thawing of plasma samples up to nine times have no effect on plasma YKL-40 levels [9,30,31]. The YKL-40 serum/EDTA plasma ratio is 1.4, and the YKL-40 levels in corresponding serum and EDTA plasma samples are correlated (rho = 0.98, p < 0.001) [30,31]. The slightly higher levels of YKL-40 in serum compared to EDTA plasma is probably caused by release of YKL-40 from activated neutrophils during the coagulation process. In this review, there will be no discrimination between YKL-40 levels in EDTA plasma and serum samples, since the levels of YKL-40 in the patients were related to EDTA plasma or serum concentrations of YKL-40 in healthy subjects. 

Plasma YKL-40 levels are relatively stable in healthy subjects during a day, a month, a year and during a period of three years. The within subject coefficient of variations including variation over time and inter-assay were 28.8% and 30.2% over a period of two and three years, and the intra-class correlation coefficients were 72.4% and 72.2% indicating reasonable reliability of plasma YKL-40 measurements. An estimated variation in plasma YKL-40 within healthy subjects including inter-assay variation suggests that an increase of more than 109% or a decrease of more than 52% in plasma YKL-40 could be considered as significant and not only a reflection of pre-analytical conditions, methodological and normal biologic variability [26]. Furthermore, plasma YKL-40 was stable in 929 healthy subjects from the Danish general population, who had plasma YKL-40 measured twice in blood samples collected with 10 years interval (Bojesen, S.E., manuscript in preparation).

## 4. YKL-40 in Different Types of Cancer

The pivotal criterion with regard to the potential clinical value of a candidate cancer biomarker is the consistency and strenght of the association between the biomarker and the outcome of the cancer of interest, and the extent to which it is an improvement on either adding to or replacing established tools. The use of plasma YKL-40 has not received Food and Drug Administration (FDA) approval for use as a biomarker in patients with cancer. It is not known whether determination of plasma YKL-40 can be useful in clinical practice, e.g., allowing clinicians to use elevated plasma YKL-40 in risk assessment of healthy subjects and patients, in treatment selection and in monitoring disease relapse or progression during and after treatment. 

**Figure 2:**
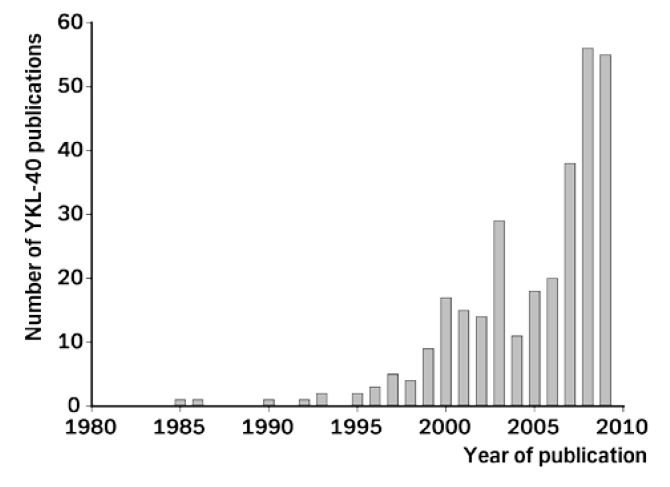
The number of publications each year regarding YKL-40.

In the next sections we will describe what has been published since the first publication 15 years ago describing plasma YKL-40 as a potential marker of prognosis in patients with recurrent breast cancer [32]. In the beginning there was little interest in plasma YKL-40 as a biomarker, but during the last few years the number of publications regarding YKL-40 has increased markedly (Figure 2). 

### 4.1. Adenocarcinoma

#### 4.1.1. Tissue

**Figure 3:**
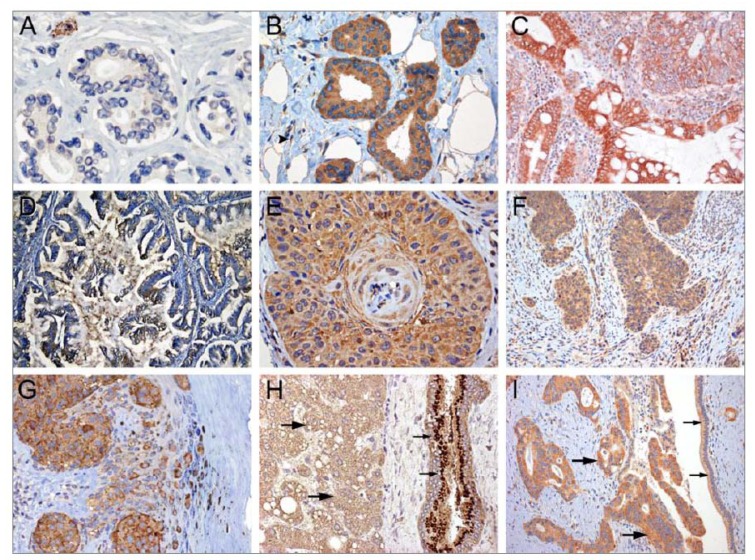
YKL-40 protein expression determined by immunohistochemical analysis in biopsies from patients with different types of cancer. (A) Normal breast epithelium with weak YKL-40 staining and strong staning in inflammatory cells; (B) Invasive ductal carcinoma of the breast with strong, diffuse cytoplasmic staining; (C) Colorectal carcinoma with strong, diffuse cytoplasmic staining; (D) Ovarian carcinoma with moderate, cytoplasmic staining; (E) Squamous cell carcinoma of the head and neck with strong, diffuse cytoplasmic staining; (F) Squamous cell carcinoma of the cervix with strong, diffuse cytoplasmic staining; (G) Melanoma with strong, diffuse cytoplasmic staining; (H) Hepatocellular carcinoma with low cytoplasmic staining (large arrows) and a bile duct with dot-like staining (in Golgi) of normal epithelial cells (small arrows); and (I) Pancreatic carcinoma with strong, diffuse cytoplasmic staining (large arrows) and low staining in normal epithelial cells (small arrows).

YKL-40 mRNA and protein expression are found in cancer tissue from breast, colon, liver, lung, ovary, pancreas, prostate, gastric and uterus [33], and in tumor associated macrophages [33,34,35,36,37,38], Figure 3 B,C,D,H,I. There are no reports of adenocarcinoma cell lines producing high levels of YKL-40 *in vitro*. This is in contrast to the very high YKL-40 production *in vitro* by the human osteosarcoma cell line MG63 [6]. Microarray gene analyses have shown that YKL-40 is overexpressed compared to normal tissue in human papillary thyroid carcinoma [39]. In breast cancer YKL-40 protein expression is high compared to normal breast tissue Figure 3A, B [40,41,42,43] and was a predictor of short disease-free survival in a small study [40]. However, this could not be confirmed in a large study of patients with primary breast cancer [43]. In colorectal, gastric, hepatocellular, pancreatic, non-small cell lung and ovarian adenocarcinoma YKL-40 protein expression is high compared to normal tissues [36,37,38,44] as illustrated in Figure 3C, D, H, I. In colorectal cancer (personal observation), non-small cell lung cancer [37], and ovarian cancer [36] the YKL-40 protein expression scores were not associated with prognosis. Whereas in gastric cancer high YKL-40 protein expression was an independent biomarker of short survival and associated with tumor invasion, lymph node metastasis and activation of Akt [38]. 

#### 4.1.2. Screening

Plasma YKL-40 has been determined in a large prospective cohort study of 8899 subjects (aged 20–95 years) from the Danish general population, the Copenhagen City Heart Study, followed for 11 years for cancer incidence and for 14 years for death. Very high plasma YKL-40 levels, *i.e.*, in the 96–100% percentile category, in subjects without known cancer predicted a 3.4 fold increased risk of gastrointestinal cancer (multifactorially adjusted for sex, age, smoking history, alcohol intake, and body mass index). Furthermore, high plasma YKL-40 levels predicted an absolute 10-year risk of gastrointestinal cancer of 14% in women and 20% in men and who were >70 years and smokers [27]. Mechanistically, this increased risk of gastrointestinal cancer may be explained either by the fact that cancer cells and tumor associated macrophages produce YKL-40, that elevated YKL-40 cause gastrointestinal cancer, or that a common factor like chronic inflammation cause both elevated plasma YKL-40 and cancer. An association between a biomarker and a disease may represent a causal relationship (causation), an increase in the biomarker as a consequence of the disease or its treatment (reverse causation), or an association that is spurious because both the biomarker and the disease are affected independently by another known or unknown factor (confounding) [45]. Studies are ongoing in relation to YKL-40 genotyping in this large cohort of 8899 subjects and we want to test the following hypotheses: (1) genetic variants in the YKL-40 gene are associated to plasma levels of YKL-40 in the general population; (2) genetic variants in the YKL-40 gene are a risk factor for future cancer; and (3) genetic variants in the YKL-40 gene are associated to certain types of cancer.

Plasma YKL-40 has also been determined in a prospective, population based study of 4987 subjects (aged 18–97 years) referred to endoscopy due to symptoms or other risk factors for colorectal cancer. 303 subjects were diagnosed with colorectal cancer [46]. Multivariate logistic regression analysis including plasma YKL-40, carcinoembryonic (CEA), age, sex, BMI, smoking, alcohol intake and co-morbidity demonstrated that plasma YKL-40 independently predicted colorectal cancer [47].

Recently, it was reported that plasma YKL-40, carbohydrate antigen 19-9 (CA-19-9) and osteopontin in combination had superior sensitivity for detection of locally advanced, stage II/III pancreatic cancer compared to CA 19-9 alone (93% *vs.* 80%) [29]. Studies are therefore ongoing to evaluate if plasma YKL-40 in combination with a panel of other biomarkers can be of value for detection of pancreatic cancer at an early stage. 

These studies suggest that plasma YKL-40 may be useful in the assessment of early detection of gastrointestinal cancer, but the results should be validated. It is also unknown if plasma YKL-40 could be used for risk assessment of other types of adenocarcinoma. 

#### 4.1.3. Prevalence of High Plasma YKL-40 Levels

**Figure 4:**
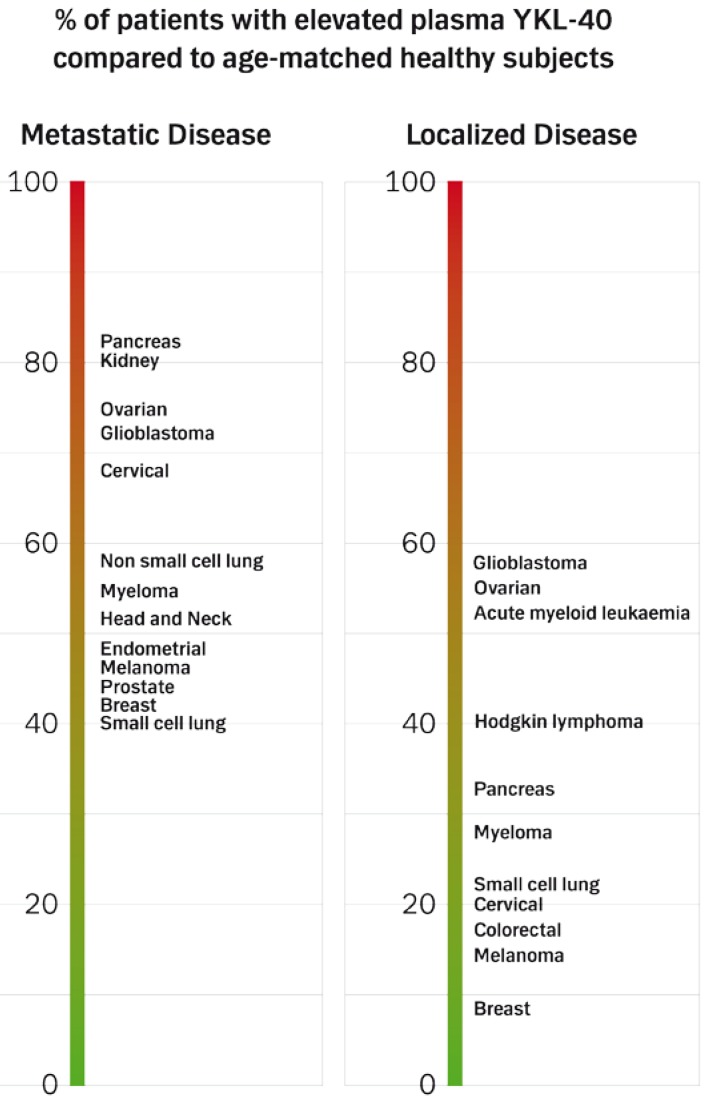
The percentage of patients with elevated plasma YKL-40 is calculated as the number of patients in the different studies with plasma YKL-40 higher than the age-adjusted 95th percentile of plasma YKL-40 in healthy subjects.

Plasma YKL-40 is elevated, defined as higher than the age-adjusted 95^th^ percentile of plasma YKL-40 in healthy subjects, in some patients with primary or advanced adenocarcinoma of the breast [33,48,49,50,51], colorectal [52,53,54], endometrial [55], lung [56], pancreas [28,29,57], prostate [58,59,60], ovary [36,61,62,63,64,65] and cervix [66]. Highest plasma YKL-40 levels are found in patients with advanced cancer. The percentage of patients with elevated plasma YKL-40 was highest in patients with metastatic pancreatic and ovarian adenocarcinoma, ~80% of these patients had elevated plasma YKL-40, Figure 4. These types of cancer are characterized by a very dismal prognosis. 

#### 4.1.4. Prognosis

High pre-treatment plasma YKL-40 is an independent prognostic biomarker of short overall survival in patients with different types of adenocarcinoma, Table 1. This was found in patients with localized or advanced breast [32,48,49,50,51], colorectal [52,53,54], endometrial [55], non-small cell lung [56], ovary [36,61,62,63,64], cervix [66] and prostate [58,59,60] adenocarcinoma both at time of first cancer diagnosis and at time of relapse. Plasma YKL-40 was independent of other routinely used biomarkers (*i.e.*, estrogen receptor status, KRAS mutation status, serum levels of human epidermal growth factor receptor 2 (HER2), CEA, carbohydrate antigen (CA) 125, prostate-specific antigen (PSA) and lactate dehydrogenase (LDH) and clinical parameters (*i.e.*, age, performance status, tumor stage, histology) when tested in multivariate Cox analysis [32,36,48,49,50,51,52,53,54,55,56,58,59,60,61,62,63,64,66]. In many of these studies, but not all, pre-treatment plasma YKL-40 was also a prognostic biomarker of recurrence- or progression-free interval. Plasma YKL-40 may therefore provide additional information of prognosis in patients with different types of adenocarcinoma.

**Table 1:** Pretreatment plasma YKL-40 is an independent prognostic biomarker of overall survival in cancer patients. The results are from multivariate Cox regression analysis including clinical characteristics and other routine prognostic variables.

Diagnosis	Number	Treatment	HR (95% CI)	P-value	Ref.
Primary breast cancer	271	Surgery + Chemotherapy	1.8 (1.0–3.1)	0.04	49
Metastatic breast cancer	100	Chemotherapy	2.6 (1.6–4.1)	0.0002	48
Colorectal cancer	603	Surgery	1.4 (1.1–1.8)	0.007	52
Metastatic colorectal cancer	155	Chemotherapy + Biologics	1.2 (1.1–1.4)	0.0045	54
Ovarian cancer, stage III	47	Surgery + Chemotherapy	4.0 (1.5–10.3)	0.005	61
Ovarian cancer, 1. relapse	73	Chemotherapy	2.3 (1.3–4.1)	0.006	62
Ovarian cancer, stage I–IV	76	Surgery + Chemotherapy	2.1 (1.4–3.2)	0.0004	36
Metastatic prostate cancer	150	Endocrine therapy	1.3 (1.0–1.7)	0.02	58
Renal cancer	58	IL-2	3.0 (1.2–7.0)	0.014	72
Small cell lung cancer	131	Chemotherapy	1.9 (1.1–3.4)	0.02	71
Metastatic NSCLC	189	Chemotherapy	1.5¤	0.0002	56
Cervical, adenocarcinoma	37	Surgery or Chemoradiation	11 (1.3–97) ^§^	0.03	66
Head- and neck cancer	138	Radiotherapy	2.2 (1.4–3.4)	0.0006	68
Melanoma I–II	234	Surgery	3.6 (1.7–7.7)	0.001	74
Melanoma IIB–III*	147	Surgery	1.8 (1.2–2.7)	0.008	75
Melanoma IV	110	IL-2, IFN	1.9 (1.2–2.8)	0.004	73
Acute myeloid leukemia	78	Chemotherapy	1.4 (1.1–1.7)	0.0002	99
Anaplastic astrocytoma	38	Surgery	2.2 (1.0–4.9)	0.05	94
Glioblastoma	75	Surgery	1.4 (1.1–1.9)	0.02	94

HR, Hazard Ratio; CI, Confidence Interval; NSCLC, Non small cell lung cancer; IL-2, Interleukin 2; IFN, Interferon-α. In most of these studies plasma YKL-40 was log transformed and treated as a continuous variable. ¤Cut-off level: 209 μg/L. ^§^Cut-off level 130 μg/L and the results of disease free survival is shown.*Blood sample collected after primary operation.

#### 4.1.5. Treatment Response

Few studies have evaluated if pre-treatment plasma YKL-40 is a predictor of treatment response. Plasma YKL-40 decreased in patients after curative operation for colorectal cancer, whereas patients with elevated plasma YKL-40 six months after operation had shorter recurrence-free interval and overall survival [53]. In patients with first recurrence of breast cancer high plasma YKL-40 predicted less responsiveness to anthracycline therapy [48], and high plasma YKL-40 was a predictor of second-line chemo resistance in patients with ovarian cancer [64]. Pre-treatment plasma YKL-40 was an independent prognostic biomarker in patients with metastatic prostate cancer treated with endocrine therapy [58]. Recently, we have found that high plasma YKL-40 in patients with metastatic colorectal cancer before 3. line treatment with cetuximab and irinotecan was associated with short progression free survival and short overall survival, independent of KRAS status [54]. 

#### 4.1.6. Monitoring

Early changes in biomarkers are investigated as potential predictors of response. They may be useful in early identification of patients unlikely to benefit, so as to spare them ineffective and potentially toxic treatment. Furthermore, monitoring plasma biomarkers in cancer patients without evidence of disease might allow for earlier detection of recurrence. There is limited information regarding changes in plasma YKL-40 during treatment of cancer patients. Increases in plasma YKL-40 during follow-up after curative resection of colorectal cancer was related to increased risk of recurrence and death [53]. During treatment of patients with metastatic colorectal cancer with 3. line cetuximab and irinotecan plasma YKL-40 as well as the ratio of updated YKL-40 levels compared to pre-treatment levels were associated with progression free survival and overall survival, and high YKL-40 values indicated poor prognosis [54]. In patients with metastatic prostate cancer treated with endocrine therapy, high plasma YKL-40 during treatment was related to short survival [59]. In a small study of patients with locally advanced pancreatic cancer increased plasma YKL-40, compared to pre-treatment levels, four to six weeks after the end of radiochemotherapy, was associated with short overall survival [57]. In patients with metastatic pancreatic cancer, an increase in plasma YKL-40 four weeks after start of chemoradiotherapy was also associated with short survival [57]. 

### 4.2. Squamous Cell Carcinoma

#### 4.2.1. Tissue

YKL-40 protein expression is high compared to normal tissue in head and neck, cervix and anal squamous cell carcinoma [66,67,68,69], Figure 3 E,F. YKL-40 protein expression was not associated with prognosis in head and neck squamous cell carcinoma [68], and has not been evaluated in cervix squamous cell carcinoma. In anal squamous cell carcinoma expression of YKL-40 was highest in keratinizing carcinoma and high expression was related to short survival [69].

#### 4.2.2. Prognosis, Treatment Response, and Monitoring

Plasma YKL-40 is elevated, defined as higher than the age-adjusted 95^th^ percentile of plasma YKL-40 in healthy subjects, in 53% of patients with head and neck cancer [69] and increased with stage in patients with cervix squamous cell carcinoma [66,67], Figure 4. High pre-treatment plasma level of YKL-40 is an independent prognostic biomarker of short overall survival in patients with head and neck and cervix squamous cell carcinoma [67,68], Table 1. It is not known if pre-treatment plasma YKL-40 is a predictor of treatment response in these patients. A longitudinal study of patients with squamous cell carcinoma of the head and neck (TNM stage III and IV) showed that lack of decrease in plasma YKL-40 two months after radiotherapy compared to pretreatment value was accompanied by short survival. Furthermore, an increase in plasma YKL-40 during follow-up in these patients predicted short survival [68]. 

### 4.3. Small Cell Lung Carcinoma

#### 4.3.1. Tissue

YKL-40 mRNA expression is not found in small cell lung cancer cells but was high in the tumor associated macrophages [70]. Human and mice small cell lung cancer cells do not produce YKL-40 *in vitro* [70].

#### 4.3.2. Prognosis, Treatment Response, and Monitoring

Plasma YKL-40 is elevated, defined as higher than the age-adjusted 95^th^ percentile of plasma YKL-40 in healthy subjects, in a subgroup of patients with localized or advanced small cell lung carcinoma [71], Figure 4. High pre-treatment plasma YKL-40 levels before chemotherapy independently predicted short survival [71], Table 1. 

### 4.4. Renal Cell Carcinoma

#### 4.4.1. Tissue

YKL-40 protein expression is found in renal cell carcinoma (personal observation, NCBI). 

#### 4.4.2. Prognosis, Treatment Response, and Monitoring

Plasma YKL-40 is elevated, defined as higher than the age-adjusted 95^th^ percentile of plasma YKL-40 in healthy subjects, in approximately 80% of patients with metastatic renal cell carcinoma [72]. Multivariate analysis including YKL-40, LDH and performance status showed that high plasma YKL-40 was an independent biomarker of short survival [72], Table 1. 

### 4.5. Melanoma

#### 4.5.1. Tissue

YKL-40 protein expression is found in melanoma cells and in tumor associated macrophages, Figure 3G. It has not been evaluated if YKL-40 expression score in melanoma is related to progression free survival or overall survival. Melanoma cells *in vitro* produce YKL-40 (personal observation). 

#### 4.5.2. Prognosis, Treatment Response, and Monitoring

Plasma YKL-40 is elevated, defined as higher than the age-adjusted 95^th^ percentile of plasma YKL-40 in healthy subjects, in 45% of patients with metastatic melanoma and in 13% of patients with stage I melanoma [73,74], Figure 4. Pre-treatment plasma YKL-40 is an independent prognostic biomarker in patients with stage I, II, and IV melanoma [73,74]. High plasma YKL-40 in patients after surgery for stage IIB-III melanoma is associated with poor survival in patients not receiving adjuvant interferon therapy [75], Table 1. In patients operated for stage I and II melanoma an association was found between high plasma YKL-40 during follow-up and short recurrence-free survival and overall survival [74]. An increase in plasma YKL-40 compared to the previous measurement was also associated to shorter overall survival in stage IIB-III melanoma patients during follow-up or treatment with adjuvant interferon [75].

### 4.6. Glioblastoma

#### 4.6.1. Tissue

*In vitro*, the U87 glioblastoma cell line [76,77] and the glioma cell lines U1242MG, U343MG and U1231MG [78] secrete YKL-40*.* Microarray gene analyses showed that YKL-40 is overexpressed, compared to normal tissue, in glioblastomas [79,80,81,82,83,84,85,86,87,88] and astrocytomas [78]. YKL-40 mRNA and protein expressions increases with glioma grade and are higher in glioblastoma than in astrocytic and oligodendroglial tumors [87,89,90,91,92,93]. Furthermore, YKL-40 expression is inversely associated with epidermal growth factor receptor (EGFR) in glioblastoma, with loss of chromosome 10q [89] and tends to be higher in astrocytomas with 10q23 loss of heterozygosity (LOH) [84,93]. High YKL-40 expression was associated with poor radiation response and short time to disease progression and death [77,84,89,90,91]. Underscoring YKL-40’s association with behaviour, it has been identified as part of a nine-gene paraffin tissue-based expression panel that most closely predicts survival in patients with glioblastoma [94]. Patients with EGFRvIII-negative/YKL-40-negative tumors had the best prognosis [91]. High YKL-40 protein expression was also associated with loss of chromosome 10q but not with amplification of EGFR. In pediatric high-grade gliomas YKL-40 overexpression is less frequent than in adult high-grade gliomas and does not correlated with survival [95]. 

#### 4.6.2. Prognosis, Treatment Response, and Monitoring

Plasma YKL-40 is elevated, defined as higher than the age-adjusted 95^th^ percentile of plasma YKL-40 in healthy subjects, in 57–72% of patients with glioblastoma [81,94], Figure 4. Baseline plasma YKL-40 was an independent prognostic biomarker of decreased survival in patients glioblastoma [94], Table 1. In patients operated for high-grade gliomas plasma YKL-40 during follow-up was lower in patients with no radiographic evidence of disease compared to patients with signs of disease, and high plasma YKL-40 during follow-up was associated with short survival [94]. However, any benefit of initiating treatment on the basis of this information is not known.

### 4.7. Hematological Malignancies

#### 4.7.1. Tissue

YKL-40 protein expression has not been evaluated in tissue from patients with leukemia and lymphoma. In myeloma, YKL-40 originates from cells in the bone marrow microenvironment surrounding the myeloma cells [96]. A few myeloma cell lines [96] and some tumor cell lines originating from immature cells of the monocytic differentiation lineage produce YKL-40 *in vitro* [4,97,98]. 

#### 4.7.2. Prognosis, Treatment Response, and Monitoring

Plasma YKL-40 is elevated in some patients with acute myeloid leukemia [99], multiple myeloma [96,100,101], and Hodgkin lymphoma [102], and high pre-treatment plasma YKL-40 is associated with short survival [96,99,100]. Patients with myeloma and high plasma YKL-40 had more severe bone destruction including increased bone resorptive activity and early progression of myeloma-related bone disease [100]. In patients with Hodgkin lymphoma and myeloma high correlations were found between plasma YKL-40 and interleukin (IL)-6 [101,102].

### 4.8. Cancer Stem Cells

#### 4.8.1. Tissue

YKL-40 protein expression is found in tissue from embryonal carcinoma, Figure 1D. There are no publications evaluating YKL-40 production by cancer stem cells. 

## 5. Plasma YKL-40 in Patients with Non-Malignant Diseases Characterized by Inflammation, Tissue Remodeling and Fibrosis

High plasma YKL-40 levels are also found in patients with non-malignant diseases characterized by inflammation, increased extracellular tissue remodeling and ongoing fibrosis. It is therefore very important to take into account any co-morbiditiy in patients with cancer, since a high plasma YKL-40 level may also originate from non-malignant cells. 

### 5.1. Inflammatory Diseases

YKL-40 is an acute phase protein because its plasma concentration increases more than 25% following an inflammatory stimulus. Plasma YKL-40 is suggested to be a biomarker of acute and chronic inflammation [25,103], including systemic low-grade inflammation [104]. In contrast to serum CRP, that is produced in the liver by hepatocytes in response to high IL-6 levels [105,106], YKL-40 is produced locally in tissues with inflammation by macrophages and neutrophils. No or low correlations are found between plasma YKL-40 and CRP, suggesting that these biomarkers reflect different aspects of the inflammatory process, and that plasma YKL-40 provides independent information of inflammation. 

Plasma YKL-40 correlated with plasma IL-6 levels in 80-year old women and men, and with serum tumor necrosis factor (TNF)α and CRP in 80-year old women. High plasma YKL-40 was also associated with a low CD4:CD8 cell ratio, and with all-cause mortality, independent of sex, smoking, BMI, chronic disease and anti-inflammatory medicine [104]. In 482 subjects without cardiovascular disease or type 2 diabetes plasma YKL-40 independently predicted cardiovascular mortality and all cause mortality [107].

#### 5.1.1. Infectious Diseases

YKL-40 is located in the specific granules of neutrophils [108] and macrophages produce YKL-40 [3,4,19]. Patients with *Streptococcus pneumoniae* pneumonia, bacteremia and sepsis have ~10-fold higher plasma YKL-40 compared to healthy subjects [109,110,111]. Human endotoxaemia, characterized by increased plasma TNFα and IL-6 levels, increases plasma YKL-40 [112]. Plasma and cerebrospinal fluid concentration of YKL-40 is associated to severity and prognosis of patients with bacterial infection, and plasma YKL-40 may add independent information of prognosis [109,110,111,113]. Plasma YKL-40 decreases faster to normal levels than CRP in patients who recover from an disease [109].

#### 5.1.2. Cardiovascular Diseases

*In vivo*, YKL-40 is expressed by macrophages in atherosclerotic plaques [114]. *In vitro* YKL-40 is produced by macrophages during late state of differentiation [3,4,19], by differentiated vascular smooth muscle cells [115,116,117,118] and by endothelial cells [119]. Recent studies suggest that plasma YKL-40 may be a potential biomarker in patients with cardiovascular disease [120,121,122,123,124,125,126,127,128]. Plasma YKL-40 increases in patients after an acute myocardial infarction [120,121] and is associated with the number of diseased vessels assessed by coronary angiography [122,123]. Plasma YKL-40 correlated with creatine kinase fraction B in non-thrombolyzed patients with acute myocardial infarction [120]. Elevated plasma YKL-40 is found in some patients with atrial fibrillation [124,125]. A study of 4298 patients with stable coronary artery disease showed that high plasma YKL-40 independently predicted cardiovascular mortality and all cause mortality [126]. It is not known if plasma YKL-40 reflects the total burden of coronary atherosclerosis or identifies a high-risk atherosclerosis phenotype with ongoing inflammation and severe atherosclerotic plaques. In a smaller study of patients with chronic heart failure plasma YKL-40 was elevated compared to healthy age-matched subjects, but was not associated with New York Heart Association (NYHA) classification, other clinical characteristics or prognosis [127].

#### 5.1.3. Diabetes

Patients with type 2 diabetes have higher plasma YKL-40 levels compared to subjects with normal glucose tolerance [9,129,130], and plasma YKL-40 is related to insulin resistance [129], fasting plasma glucose, and plasma IL-6 [130], but not to serum CRP [129] or obesity [130]. Patients with type 1 diabetes have elevated plasma YKL-40 compared to healthy subjects, and increasing plasma YKL-40 levels are associated with increasing levels of albuminuria [131]. It is suggested that plasma YKL-40 is an emerging biomarker in patients with cardiovascular disease and diabetes [132]. 

#### 5.1.4. Rheumatic Diseases

YKL-40 is produced by arthritic chondrocytes [7,133,134], synoviocytes and fibroblast-like synovial cells [7,135,136], and by macrophages in inflamed synovial tissue [133,137]. In patients with rheumatoid arthritis YKL-40 protein is expressed by CD16+ monocytes with a dim expression of CD14 [137]. This phenotype can differentiate from classic CD14++ monocytes by maturation *in vitro* and is considered as pro-inflammatory with properties of tissue macrophages and are a source of TNFα [138]. Patients with very active rheumatoid arthritis have elevated plasma YKL-40 [9,31,133,139,140,141,142,143,144,145,146,147] compared to healthy subjects and patients with inactive rheumatoid arthritis. Plasma YKL-40 is related to clinical parameters of disease activity and serum CRP, and in a few studies also with progression of joint destruction [139,140]. However, plasma YKL-40 did not provide better clinical information of disease activity and prognosis in patients with rheumatoid arthritis compared to serum CRP. 

Few patients with osteoarthritis have elevated plasma YKL-40 [133,148], and only if they have severe synovitis of large joints like a knee joint [133]. 

YKL-40 is expressed by macrophages and giant cells in arteritic vessels [119]. Patients with giant cell arteritis have elevated plasma YKL-40 at time of diagnosis, but during treatment with glucocorticoids plasma YKL-40 was not related to disease activity and serum CRP [119]. 

#### 5.1.5. Lung Diseases

YKL-40 is expressed by macrophages in bronchial-biopsy specimens and cytospin of broncho-alveolar lavage from patients with asthma [149] and chronic obstructive pulmonary disease (COPD) [150], and in sarcoid lesions of patients with pulmonary sarcoidosis [151]. YKL-40 is also produced by mast cells [24,43]. YKL-40 concentrations in plasma and bronchoalveolar lavage are higher in smokers with COPD than smokers without COPD [150] and associated with airflow obstruction and impaired diffusion lung capacity. Plasma YKL-40 levels are elevated in some patients with asthma compared to healthy subjects, and is associated with the severity of asthma measured by clinical variables, including FEV1, and with thickness of the subepithelial basement membrane in biopsy specimens of the lung [149]. YKL-40 levels in plasma and bronchoalveolar-lavage-fluid increase 24 hours after allergen challenge in patients with allergic asthma, and correlate with eosinophil counts 24 hours after allergen challenge [149,152]. Plasma YKL-40 is higher in patients with pulmonary sarcoidosis compared to healthy subjects and correlated with serum angiotensin-converting enzyme [151]. Patients with systemic sclerosis and elevated plasma YKL-40 have poor prognosis and often die due to extensive interstitial or vascular fibrosing processes [153,154]. It has therefore been suggested that plasma YKL-40 is a potential new biomarker in patients with inflammatory lung diseases [103,155].

#### 5.1.6. Inflammatory Bowel Diseases

Elevated plasma YKL-40 levels are found in patients with active inflammatory bowel disease [142,156,157,158,159]. In patients with ulcerative colitis plasma YKL-40 correlated with serum CRP and a disease activity score [156,158]. In patients with Crohn’s disease both low relations between plasma YKL-40 and disease activity score [158], and high relations between plasma YKL-40 and activity score [156] are reported. Patients with inflammatory bowel disease and joint involvement have higher plasma YKL-40 than patients without joint involvement [157]. Patients with Crohn’s disease and stenotic disease have higher plasma YKL-40 than patients with non-stenotic disease and plasma YKL-40 was independent of other clinical parameters [156,159]. Thus, plasma YKL-40 may reflect ongoing fibrogenesis and may be a risk factor in patients with Crohn’s disease. 

### 5.2. Liver Fibrosis

High YKL-40 protein expression is found in fibrotic liver tissue from patients with alcoholic liver disease and chronic hepatitis C virus infection [160,161]. Hepatocytes do not express YKL-40 protein and no expression is found in normal liver tissue except in mesenchymal structures of the portal tract. It is most likely that hepatic stellate cells, leucocytes and macrophages produce YKL-40 in the fibrotic liver [160,161]. In biopsies with chronic active hepatitis C virus YKL-40 protein expression was found in areas with piecemeal necrosis, but not in lymphocytes. 

In 8899 subjects from the general population increasing plasma levels of YKL-40 were found with increasing alcohol intake [27]. Plasma YKL-40 is elevated in most patients with moderate to severe liver fibrosis and cirrhosis, independently of disease etiology, and may provide new information of ongoing fibrogenesis in the liver [160,161,162,163,164,165,166,167,168,169,170,171,172,173,174,175,176,177,178]. In patients with alcoholic liver disease elevated plasma YKL-40 is related to liver fibrosis and inflammation in the liver [160,161,164], and patients with very high plasma YKL-40 have shorter survival than patients with normal plasma YKL-40 [163]. Plasma YKL-40 predict cirrhosis (Ishak 5/6) in patients with chronic hepatitis C, but was not included in the final three-variable model consisting of serum hyaluronan, tissue inhibitor of metalloprotinases (TIMP)-1 and platelet count [171,172]. In children, plasma YKL-40 could not differentiate patients with advanced liver fibrosis from those with mild fibrosis [173]. 

## 6. What Is the Function of YKL-40?

The biological functions of YKL-40 in cancer are unknown. YKL-40 may play different roles depending upon the cell types and environment. YKL-40 probably has a role in proliferation and differentiation of malignant cells and protects the cells from apoptosis. YKL-40 also stimulates angiogenesis and has an effect on extracellular tissue remodeling, and YKL-40 regulates fibroblast activity and increases the degree of fibrosis surrounding cancer cells, although *in vivo* proofs are yet to be obtained [25,34,35]. Membrane receptors of YKL-40 are not described. YKL-40 induced activation of intracellular signal-transduction pathways suggests that it interacts with signaling components on the cell membrane [179,180,181]. A thorough understanding of the pathobiology of a putative novel biomarker is not essential regarding its diagnostic or prognostic performance as a biomarker. However, pathophysiological information is very useful in providing support for the validity of the clinical observations, as well as direct research that pertains to possible diagnostic and therapeutic applications. 

### 6.1. Single Nucleotide Polymorphism (SNP)

Studies are ongoing to describe genetic variants of YKL-40 in the general population. The level of YKL-40 expression may in some cases be genetically regulated. The most tested SNP-131 C/G in the promoter region of the YKL-40 gene did not differ between patients with glioblastoma and controls, and no correlation was found between this genotype and YKL-40 expression in glioblastoma samples [182]. There was no difference in survival between the CC, CG, and GG glioblastoma patients despite the few GG patients tended to have a longer survival [182]. In other diseases SNP-131 C/G is associated with: (1) elevated plasma YKL-40 levels in patients with asthma, bronchial hyper responsiveness and pulmonary function in the Hutterites [183]; and (2) plasma YKL-40 levels and the severity of hepatitis C virus induced liver fibrosis [162]. An association is found between schizophrenia and haplotypes within the promoter region of the YKL-40 gene including SNP-131 C/G [184,185,186]. A meta-analysis suggests that the genetic variants in the YKL-40 gene have ethnic heterogeneity and confer a susceptibility to schizophrenia in Asian populations [186]. In patients with rheumatoid arthritis no association was found to SNP-131 C/G [187]. SNP-329 G/A in the YKL-40 gene are related to plasma YKL-40 levels in healthy subjects but not in patients with sarcoidosis [188]. In Danish patients with type 2 diabetes [189] and asthma and atopy [190] none of the reported SNPs or haplotype blocks of the YKL-40 gene were associated with these diseases. 

### 6.2. Growth Factor

Studies of embryonic and fetal cells [5], macrophages [3,4,19,191,192,193] and fetal chondrocytes [5,194,195,196] suggest that YKL-40 is a proliferation and differentiation marker. YKL-40 is strongly expressed in the three germ layers of human embryos and exists in two different isoforms in early human fetal tissue derivatives of ecto-, meso- and endoderm. At the cellular level YKL-40 protein expression was high in embryonic and fetal tissues characterized by rapid proliferation and marked differentiation, and in tissues undergoing morphogenetic changes [5]. We have also observed that YKL-40 is produced by undifferentiated human embryonic stem cells and their progenitors in differentiating human embryonic stem cells colonies [23]. Embryonal carcinoma also expresses YKL-40 protein and studies are ongoing to test if YKL-40 is produced by different types of cancer stem cells. 

### 6.3. Protection Factor

YKL-40 expression increases in human glioblastoma cells following stress stimuli such as hypoxia, serum depletion, ionizing radiation and chemotherapy [76]. The response is late, 24 hours to 72 hours after stimuli, indicating that YKL-40 is a secondary response downstream of other mechanisms. Astrocytes transfected with YKL-40 have increased resistance to serum depletion and radiation as well as increased invasion potential [84]. Tumor growth of the human U87 glioblastoma cells as xenografts on nude mice is delayed by treatment with monoclonal antibodies against human YKL-40 [197], suggesting that YKL-40 may be a potential cancer target. *In vivo*, in glioblastoma cells, a positive association between YKL-40 and activated AKT1 pathways and MAPK intermediates was found [91]. It was hypothesized that YKL-40 as a secreted protein may serve as an extracellular signal, inducing increased downstream activity of Ras [198], or may be a surrogate measurement of Ras/PI3-K activation [91]. 

YKL-40 initiates PI-3K signalling pathways and phosphorylation of AKT [179,180,181] and may be an anti-apoptotic protein [199]. YKL-40 is also called "breast regression protein (Brp-39)" [15], since it is induced in mice mammary epithelial cells a few days after weaning. Mammary involution involves programmed cell death. It is suggested that YKL-40 utilizes a chitin oligosaccharide binding ability while participating in the various signal transduction pathways that leads to apoptosis of the regressing cells and that YKL-40 is a protective signalling factor determining which cells are to survive the high tissue remode that occurs during involution [13]. 

### 6.4. Angiogenesis

A recent study has identified YKL-40 as a promoter of angiogenesis in cancer, including activating the MAPK/ERK pathway in endothelial cell [200]. Transfection of the human glioblastoma cell line U87 with short-interfering RNA against vascular endothelial growth factor (VEGF-A) and implantation on a chick chorio-allantoic membrane resulted in an up-regulation of YKL-40 [77]. This suggests a role of YKL-40 in regulating response of cancer cells to hypoxia. YKL-40 is also synthesized by vascular smooth muscle cells [8,115,116,117,118], stimulates migration of endothelial cells [117], and promotes vascular smooth muscle cell attachment, spreading and migration [118] suggesting a function in angiogenesis and thereby playing a role in the growth of the tumor [201]. 

### 6.5. Inflammation

YKL-40 plays a pathogenetic role in colitis by enhancing the adhesion and invasion of bacteria on/into colonic epithelial cells, and leads to exacerbations of intestinal inflammation [202,203,204]. YKL-40 is an autoantigen in rheumatoid arthritis and may have a fundamental role in the pathophysiology of rheumatoid arthritis [137,205,206,207,208,209,210,211]. Studies of patients with asthma suggest that YKL-40 has a role in the innate immune response [149,212]. A study of Brp-39 (the mouse homologue of YKL-40) in Brp-39^−/−^ mice, YKL-40 transgenic mice, and mice that lack Brp-39 and produce YKL-40 only in their pulmonary epithelium found that Brp-39/YKL-40 has several important functions: (1) it is an inhibitor of macrophage, T-cell, and eosinophil death receptor-mediated apoptosis/cell death and is associated with augmented protein kinase B (PKB)/AKT phosphorylation and Faim 3 induction; (2) it plays a role in the pathogenesis of the IL-13 effector responses that generate inflammation and air-way remodelling; (3) it stimulates accumulation and activation of pulmonary dendritic cells and macrophages; and (4) it is involved in antigen-induced sensitization and IgE induction [199].

### 6.6. Tissue Remodeling and Development of Fibrosis

The stroma around the periphery of solid tumors has many similarities with granulation tissue such as that found in wound-healing or inflammation, and regulates essential aspects of tumor proliferation, cell death, progression, matrix remodeling and angiogenesis, and subsequently promotes tumor growth and progression of metastatic disease [213,214,215,216,217,218,219,220,221,222]. YKL-40 is a proliferation factor of fibroblasts [14,179] and acts synergistically with IGF-1 [179]. YKL-40 secreted by cancer cells and inflammatory cells surrounding and infiltrating the tumor may play a role in proliferation, activation and differentiation of the fibroblasts/myofibroblasts surrounding the tumor. YKL-40 could thereby influence development of the prominent desmoplastic stroma seen in both primary cancer and metastatic sites. This phenomenon, termed stromal reaction, includes activation of fibroblasts and myofibroblasts transformation, inflammation, secretion of cytokines, matrix proteins and metalloproteinases, and angiogenesis. All play a role in cancer development and metastatic potential, affecting the proliferation, differentiation, invasion or regression of cancer cells, particularly in cancers of epithelial origin [221,223,224,225,226]. 

YKL-40 is regulated in chondrocytes by TNFα [180,181] and requires sustained activation of NF-κB [181], which controls cell survival by regulating cell proliferation, growth arrest and death [214]. YKL-40 initiates MAP kinase and PI-3K signalling cascades in fibroblasts leading to phosphorylation of the extracellular signal-regulated kinase (ERK)-1/2 MAP kinase and PKB/AKT-mediated signalling cascades, which are associated with the control of mitogenesis [179,180,181]. Stimulation of articular chondrocytes or skin fibroblasts with IL-1 or TNFα in the presence of YKL-40 results in reduction of both p38 and SAPK/JNK phosphorylation, and YKL-40 suppresses the cytokine-induced secretion of several metalloproteinase and the chemokine IL-8 [181]. This suggests that YKL-40 may play a protective role in inflammatory environments, limiting degradation of the extracellular matrix and thereby controlling tissue remodelling. 

YKL-40 binds collagen type I, II and III and modulates the rate of type I collagen fibril formation [227]. YKL-40 also binds chitin, but has no chitinase activity [7,19,21] due to amino acid substitution in the active site of chitinases [7,19]. YKL-40 contributes to chondrocyte differentiation by inducing the transcription factor SOX9 and type II collagen expressions, and the induction of SOX9 depends on ERK1/2 and PI3K activities, but not on p38 and JNK MAPK [228]. Vertebrates in an embryonic stage use short chito-oligosaccharides as primers for the synthesis of hyaluronan [229,230,231]. YKL-40 has heparin and hyaluronan binding motifs [20] and may bind to cell surface receptors such as heparin sulphate proteoglycans and may recognize hyaluronan or its precursor as a substrate in the extracellular matrix and interfere with the synthesis and local concentrations of hyaluronan [20]. YKL-40 may influence the effects of high hyaluronan in tissues, e.g., the extent of cell adhesion and migration during the tissue remodelling processes that take place during metastasis, inflammation, fibrosis, and atherogenesis [232,233]. Furthermore, YKL-40 secreted from macrophages in adipose tissue inhibits degradation of type I collagen and increases the rate of fibril formation of type I collagen [234].

## 7. Plasma YKL-40—A New Cancer Biomarker?

The first paper regarding plasma YKL-40 levels in cancer patients was published in 1995 [32]. Unfortunately, it is still unknown whether determination of plasma levels of YKL-40 can be useful in clinical practice. The pivotal criterion with regard to the potential clinical value of a candidate cancer biomarker is the consistency and strenght of the association between the biomarker and the outcome or disease of interest, and the extent to which it is an improvement on either adding to or replacing established tools. The clinical utility of the biomarker should also be demonstrated by showing that result-based decisions improve patient outcome [235,236,237,238]. 

It needs to be determined if routine measurement of plasma YKL-40 in patients with cancer can provide useful clinical information for risk assessment, for treatment selection or for monitoring patients after intervention with different therapeutics. At present plasma YKL-40 can not be regarded as a new cancer biomarker, and FDA has not yet approved the use of plasma YKL-40 as a biomarker in patients with cancer or any other disease. Assessment of the clinical potential of a novel biomarker can be structured around three fundamental questions [239]:

- Can the clinician measure the biomarker and is the method specific, sensitive, fast and cheap?
- Does the biomarker level add new information of the disease?
- Does the biomarker level help the clinician to treat patients?

Plasma levels of YKL-40 can be measured accurately and fast with relatively low cost, but it is not known if plasma YKL-40 in an individual cancer patient is so reliable that it can be used to make clinical decisions that will improve outcome of the patient. 

In order to propose guidelines on how promising tumor markers progress from the laboratory into the clinic, Hayes introduced the “Tumor Marker Utility Grading System” [236,237]. According to this system a number of validation requirements have to be fulfilled before plasma YKL-40 can be considered to be a cancer biomarker. Most of the plasma YKL-40 biomarker studies are retrospective and the blood samples were not collected with the intent of testing the value of plasma YKL-40 as a diagnostic, prognostic, predictive and monitoring biomarker. There are therefore many limitations to the conclusions made from the present studies of plasma YKL-40 as a biomarker. According to the “Tumor Marker Utility Grading System” guidelines [236,237], the next step would be to launch an appropriate prospective study where the benefit of using plasma YKL-40 levels in the clinical decision-making process is assessed. Endpoints should include overall survival, disease-free survival, quality of life and cost-effectiveness. The study could be designed either as a single, highly-powered, prospective, controlled study with the primary objective of testing plasma YKL-40 level as a “prognosticator” or a similar prospective study where the primary goal could be the testing of a therapeutic hypothesis and secondly testing plasma YKL-40 as a biomarker. Such studies will hopefully be designed. Recently, it has been reported that YKL-40 in combination with a panel of biomarkers can give important information in preclinical drug development [240].

The term “cancer biomarker” embraces a spectrum of molecules of widely divergent characteristics, but sharing an association with malignancy that facilitates their application in the clinical detection (screening, diagnosis) and management (prognosis, monitoring) of cancer patients [238]. Table 2 summarizes what is presently known about plasma YKL-40 as a potential new cancer biomarker. YKL-40 is neither organ nor tumor specific. Plasma YKL-40 levels may have a role in screening for gastrointestinal cancer or identifying patients at risk [27,47]. Elevated plasma YKL-40 levels, compared to age-matched healthy subjects, are found in patients with 16 different types of cancer. Eighteen studies of 13 different types of cancer have shown that high pretreatment plasma YKL-40 is related to poor prognosis. Highest plasma YKL-40 levels are found in patients with metastatic cancer and plasma YKL-40 provides independent information of recurrence- and progression free survival and of overall survival. The potential values of plasma YKL-40 as a biomarker in monitoring and screening of cancer need more studies, and its value in combinations with other biomarkers has to be determined. It is likely that plasma YKL-40 should be combined in panels of biomarkers for optimal clinical use, since most biomarkers will probably individually lack optimal sensitivity and specificity. A study of patients with pancreatic cancer suggests that this may be useful [29]. 

**Table 2:** Is plasma YKL-40 a new cancer biomarker?

**Tumor specific?**
No—YKL-40 is also produced by non-malignant cells, e.g., inflammatory cells.
**High specificity for cancer?**
No—Plasma YKL-40 is also elevated in patients with diseases characterized by acute or chronic inflammation, tissue remodeling and fibrosis; *i.e.*, co-morbidity shall always be considered in cancer patients with high plasma YKL-40.
**High sensitivity for cancer?**
No—Plasma YKL-40 is only elevated in a subgroup of cancer patients.
**Useful for screening?**
???—Plasma YKL-40 is elevated many years before a gastrointestinal cancer is diagnosed; more studies are needed.
**Reflect poor prognosis?**
Yes—High plasma YKL-40 reflects poor prognosis and is independent of other routinely used biomarkers.
**Predictor of treatment response?**
???—High plasma YKL-40 reflects poor treatment response in some patients; more studies are needed.
**Useful for monitoring?**
???—High plasma YKL-40 may reflects disease progression; more studies are needed.

However, YKL-40 is not cancer specific, and high plasma YKL-40 levels are also found in patients with diseases characterized by inflammation, tissue remodelling and fibrosis. Co-morbidity should therefore always be considered in cancer patients. 

## 8. Future

Much more basic research related to the function and regulation of YKL-40 is needed and many fundamental questions regarding YKL-40 remain to be answered. The biological functions of YKL-40 are unclear and its role in cancer development and the mechanisms by which it reflects cancer aggressiveness and cancer progression are poorly understood. The mechanisms by which stimuli lead to increased expression and synthesis of YKL-40 are unknown. It is likely that YKL-40 has a receptor, but it has not yet been identified. YKL-40 is produced by embryonic stem cells and embryonal carcinoma, and it probably has important roles in both embryonic and fetal growth and in pathological growth like cancer. It deserves to be tested if cancer stem cells produce YKL-40, and if the protein is related to metastatic potential in combination with a function in inflammation, angiogenesis, apoptosis and tissue remodeling processes and in pathological conditions leading to fibrosis. 

Future focused translational research projects combining basic and clinical research are needed in a joint effort to answer the questions:

- Is plasma YKL-40 a useful clinical biomarker in patients with cancer?
- Is YKL-40 a target for development of new cancer therapeutics?

and with close collaborations between multidisciplinary teams including surgeons, oncologists, pathologists, biochemists, tumor biologists, molecular biologists, biotech companies and the pharmaceutical industry. Without such collaboration it is unlikely that these two questions will ever be answered. 
